# Development and validation of liquid chromatography method for simultaneous determination of multiclass seven antibiotic residues in chicken tissues

**DOI:** 10.1186/s13065-022-00797-y

**Published:** 2022-02-21

**Authors:** Aynalem Lakew, Teshome Assefa, Meseret Woldeyohannes, Negussie Megersa, Bhagwan Singh Chandravanshi

**Affiliations:** 1 Ethiopian Public Health Institute, P. O. Box 1242/5654, Addis Ababa, Ethiopia; 2 Department of Chemistry, College of Natural and Computational Sciences, Addis Ababa University, P. O. Box 1176, Addis Ababa, Ethiopia

**Keywords:** Antibiotic residues, Food safety, Food contaminants, Chicken tissue, Liquid chromatography

## Abstract

**Background:**

Antibiotics are routinely used on poultry for therapy and prevention of diseases and to enhance animal growth. The objective of this study was to develop and validate a sensitive and reliable liquid chromatography with UV detection (LC-UV) method for the simultaneous determination of seven multiclass antibiotic residues (amoxicillin, ampicillin, penicillin, sulfamethoxazole, gentamicin, ciprofloxacin, and erythromycin) in chicken tissues.

**Methods:**

The liquid chromatography method with UV detection was optimized for complete separation of the seven selected antibiotic compounds with reversed phase and isocratic elution using Hypersil BDS-C18 (3 µm, 100 mm × 4 mm) column. The mobile phase consisted a ratio of 0.05 M Na_2_HPO_4_, acetonitrile and methanol (70:10:20), at UV absorption wavelength of 230 nm. The column thermostat was set at 40 °C, the mobile phase flow rate was 1 mL min^−1^, and the injection volume was 20 μL.

**Results:**

All the seven standard compounds were eluted within 14 min. The results for: linearity, precision, sensitivity, accuracy, specificity, decision limit (CCα), detection capability (CCβ), suitability and method robustness were validated according to the criteria of Commission Decision 2002/657/EC guidelines. Calibration plot correlation coefficients ranged from 0.9983 to 0.9998 and the percent relative standard deviations for repeated analysis were below 5% indicating acceptable method precision. The limits of detection (LODs) and quantification (LOQs) ranged from 0.098–0.255 μg kg^−1^ to 0.297–0.574 μg kg^−1^, respectively. The accuracy study yielded recoveries in the ranges 98.1–107% for the pure compounds and 94.0–102% for the spiked drug free chicken tissue samples.

**Conclusions:**

The method was found to be appropriate for simultaneous determination of five different classes of seven antibiotic residues in chicken tissues. Furthermore, this is the first instance for the simultaneous determination of seven multiclass, multi-residues analysis using LC-UV from chicken tissue samples. This is a cost-effective and alternative method with simple instrumentation approach for laboratories that lack highly specialized state-of-the-art instrumentation.

## Background

Antibiotics have been routinely used in veterinary medicine and agriculture since the 1950s [1]. The use of antibiotics has not been limited to treating sick animals, but also used as feed additives in the animal husbandry to increases animal growth and their productions [2–5].

In poultry production amoxicillin is used to combat respiratory and other bacterial infections [6], ampicillin for promotion of growth, feed efficiency and stimulation of egg production [7], assistance in relieving stress [8], and rehydration of livestock [8]. It is estimated that 80% of all food-producing animals receive medication for part or most of their lives [6].

Slaughtering the chicken or laying eggs can leave pharmaceutical residues in the tissues and eggs at concentrations that can be harmful to the human health [9, 10]. Human exposure to the veterinary medicinal products through the uptake of their residues in poultry products has been linked to the development of allergic reactions in the hypersensitive individuals (e.g., penicillin) [6]; carcinogenicity (e.g., sulfamethazine, oxytetracycline, and furazolidone) [11]; hepatotoxicity, reproductive disorders, bone marrow toxicity (e.g., chloramphenicol) [6]; estrogenic, neurotoxicological effects, allergies (e.g., penicillin) [12] as well as induction and generation of resistant strains of human pathogenic bacteria [13].

World Health Organization (WHO) [14], Food and Agriculture Organization (FAO) [15] and Codex Alimentarius Commission (CAC) [16] have set standards for acceptable daily intake (ADI) and maximum residue limits (MRLs) in foods. According to commission regulation (EU), the antibiotic residue MRLs in chicken tissue are: for ciprofloxacin 100 μg kg^−1^, erthyromycin 200 μg kg^−1^ and 50 μg kg^−1^ for each of amoxicillin, ampicillin, penicillin G and gentamicin [17]. To ensure the food safety from contamination with antimicrobial residues, its use must be monitored strictly and therefore, sensitive analytical methods are essential to assay these compounds in complex matrices.

Microbiological or screening methods and chromatographic methods have been described for monitoring and detecting antibiotic residues [18]. Recent reviews described the analytical qualitative and quantitative methods that have been developed during the past decade for some antibiotic residues (microbiological approaches, biosensors, and chromatographic methods) [19]. Analytical techniques including liquid chromatography (LC) [20] and gas chromatography (GC) [21] are commonly employed for the separation and determination of compounds in mixtures. The low solubility in organic solvents, insufficiently volatile or too thermally unstable property of some antimicrobials has made it more difficult and time consuming to develop procedures and to determine antibiotic residues using GC [22].

Recent development on the new analytical strategies and confirmatory methods for residue analysis of animal products are based on the liquid chromatography (LC) and hyphenated techniques such as liquid chromatography-tandem mass spectrometry (LC–MS/MS) [23] and liquid chromatography-quadrupole time of flight mass spectrometry (LC-QToFMS) [24]. Liquid chromatography-triple quadrupole-mass spectrometry (LC-QqQ-MS) [25], time-of-flight (ToF) or high-resolution mass spectrometry (HR-MS) resulted in an enormous improvement of analytical parameters such as sensitivity and lower detection limits. Consequently, these enabled the analysts to detect multiclass and multi-component antibiotics in complex biological samples with high sensitivity, specificity and robustness [26, 27].

However, the implementation to control the antimicrobial residues is still limited in developing countries like Ethiopia due to lack of the complex laboratory equipment and the high cost required. The way to improve cost-effectiveness is to maximise the number of analytes that may be determined by a single procedure. Multi-residue detection methods using the available single instrument is an alternative for the determination and confirmation of many antibiotic residues simultaneously by LC-UV. Developing such an analytical method is more cost-effective than changing parameters for each analyte for the analysis of real samples.

To the best of our knowledge there is no single LC method reported for the simultaneous determination of the selected seven antibiotic compounds of five different therapeutic classes including three (β-lactams), amoxicillin (AMOX), ampicillin (AMPI), penicillin G (PEN G), (sulphonamides), sulfamethoxazole (SULFA) (aminoglycosides), gentamicin (GENTA) (fluoroquinolones), ciprofloxacin (CIP) and (macrolide), erthyromycin (ERYTHRO) in chicken tissue samples. This study attempts to develop a simple, accurate, precise and stable analytical chromatographic method, which can separate and determine the seven selected antibiotic drugs simultaneously in a single optimized method in chicken tissues. The proposed method has been developed and validated as per the Commission Decision 2002/657/EC guidelines [28]. The developed method is suitable for laboratories that are not equipped with highly specialized state-of-the-art instrumentation.

## Materials and methods

### Chemicals and reagents

Negative concentration chicken tissue control (Charm Scientific) was kind donation from Ethiopian Public Health Institute (EPHI). Antibiotic standard compounds (assigned purity ≥ 99%) listed in Table 1 were a kind donation from Ethiopian Food and Drug Administration (EFDA). All the standard solutions were prepared in HPLC grade methanol (> 99%) and HPLC grade acetonitrile (> 99%) were from Merck (Germany). Disodium hydrogen phosphates (Na_2_HPO_4_) (> 99%) and orthophosphoric acid (H_3_PO_4_) (> 85%) were from Sigma-Aldrich (USA). Double distilled deionized water used throughout the study was purified using Water Still, 4 LPH, Double distilled, 240 VAC, 50/60 Hzfrom Stuart Aquatron (USA).

**Table 1:** Physicochemical properties and chemical structures of the selected antibiotics commonly used on poultry production [29, 30]

Class	Compound molecular formula and weight	Chemical structure	Solubility in water (mg/mL)	Log *K*_ow_	p*K*_a_
β-Lactams	Amoxicillin, C_16_H_19_N_3_O_5_S, 365.4 g mol^−1^		3.4	0.87, 0.97	2.4, 2.8, 7.2
	Ampicillin, C_16_H_19_N_3_O_4_S, 349.4 g mol^−1^		10.1	1.45	2.53, 2.7 7.3
	Benzylpenicillin (penicillin G), C_16_H_18_N_2_O_4_S, 334.4 g mol^−1^		0.2	1.85	2.7, 2.8
Fluoro-quinolones	Ciprofloxacin C_17_H_18_FN_3_O_3_, 331.3 g mol^−1^		36	0.4	3.01, 6.38, 8.70
Aminoglycoside	Gentamicin C_21_H_43_N_5_O_7_, 477.6 g mol^−1^		100	-1.88	8.2
Macrolides	Erythromycin C_37_H_67_NO_13,_ 733.9 g mol^−1^		2	3.06, 2.48	8.88, 8.9
Sulfonamides	Sulfamethoxazole C_10_H_11_N_3_O_3_S, 253.3 g mol^−1^		0.61	0.89, 0.48	1.85, 5.6

K_ow_: the octanol–water partition coefficient

pK_a_: acidity constant

### Instruments and equipment

Shimadzu LC-20ad prominence equipped with quaternary pump, and dual wavelength UV detector, column oven and auto sampler (Shimadzu Corporation, Kyoto, Japan) and analytical column Hypersil BDS-C18 (3 µm, 100 mm × 4 mm) (Thermo Fisher Scientific, Phenomenex, USA) were used for chromatographic separation; data acquisition and processing were accomplished with LC solution software. Syringe membrane filters 0.45 μm Millex-HN (Millipore, Bed-ford, MA, USA) for filtration of standards and samples; centrifuge, AX-320 (Tomy Seiko Co., Tokyo, Japan); vortex mixer, Vortex-Genie 2 (Scientific Industries Inc., Bohemia, New York, USA) and ultrasonic machine, B5510J-DTH (Branson, Danbury, CT) were also used. The pH values of the mobile phases were measured using a Hanna instruments pH meter (Hanna Instruments Inc., Cluj-Napoca Jud, Cluj, Romania); vacuum filtration assembly (Millipore filter cellulose nitrate gridded with 0.22 μ size and 47 mm diameter) attached with vacuum pump and glass support, NS 40/35 joints from Sigma-Aldrich (USA) were used for HPLC solvent purification. Flask (Pyrex), volumetric, class A, w/Pyrex standard taper stopper, 1 mL, Corning 5640-1 (Beijing, China) and R-100 rotary evaporator from Buchi Labortechhnik AG (Switzerland) were used for evaporation of solvents.

### Chromatographic conditions

Chromatographic separation of the selected seven antibiotic standard compounds was achieved with analytical column, Hypersil BDS-C18 (3 µm, 100 mm × 4 mm) (Phenomenex, USA) in reversed phase and isocratic elution. Individual antibiotic compound and a mixture of standards were detected at 230 nm using UV detector. The mobile phase contained a combination of 0.05 M Na_2_HPO_4_, acetonitrile and methanol (70:10:20) at pH 8. The mobile phase was pumped from the reservoir to the column at a flow rate of 1 mL min^−1^. The column thermostat was set at 40 °C and the injection volume was 20 μL. All the seven standard compounds were eluted within 14 min.

### Preparation of standard stock solution

The stock standard solutions (1000 μg mL^−1^) were prepared individually for the selected seven drug standards by weighing 10 mg of reference standard substances and dissolving them in 1:1 mL of methanol:deionized water (v/v) in 10 mL volumetric flask. The stock solutions were stored at −18 °C and prepared fresh every 1 month. Mixed stock solutions of the seven antibiotic standards (200 μg mL^−1^) and the series of working standard solutions for the method development were prepared daily with a mobile phase dilution.

All the standard solutions prepared for the LC were filtered through a 0.45 μm nylon syringe membrane filter before use. The mobile phase was filtered through the Millipore glass filter (Millipore filter cellulose nitrate gridded with 0.22 μ size and 47 mm diameter) assembly attached with vacuum pump and was sonicated with ultrasonic machine, B5510J-DTH (Branson, Danbury, CT), for 30 min before pumping into HPLC system for degassing.

### Calibration standard solutions

Five calibration standard solutions of 0.05, 0.5, 1, 1.5 and 2 μg mL^−1^ were prepared from their working stock solutions (200 μg mL^−1^), by transferring the appropriate aliquot and bringing the total volume to 10 mL using mobile phase dilution for the method development. Instrument blanks were prepared by placing a portion of the acetonitrile/methanol (10:20 ratio) solution used for sample reconstitution in an amber auto sampler vial for the instrumental analysis.

### Sampling

Large-scale commercial poultry farms, village broiler and chicken egg producers are distributing the poultry products within the urban and peri-urban area of the capital city of Ethiopia, Addis Ababa. Across the city, poultry markets of various structures ranging from day-old chicks, retail eggs, slaughtered and frozen chicken markets as well as live poultry wholesaler do exist. Ten chicken samples (comprising 10 pieces each of liver, kidney and muscle) purchased from randomly selected supermarkets in Addis Ababa in October 2020 for antibiotic residue analysis. The samples were slaughtered and market-ready matured chickens that were prepared to vend for consumers in the supermarkets. After arrival at the laboratory, the samples were stored at −20 °C until analysis. Addis Ababa (AA) was chosen as sampling site because of most of the markets, supermarkets and consumers are found in this city (since AA has much higher population compared to the other cities, and have more consumption of chickens than other cities).

### Sample preparation

**Fig. 1:**
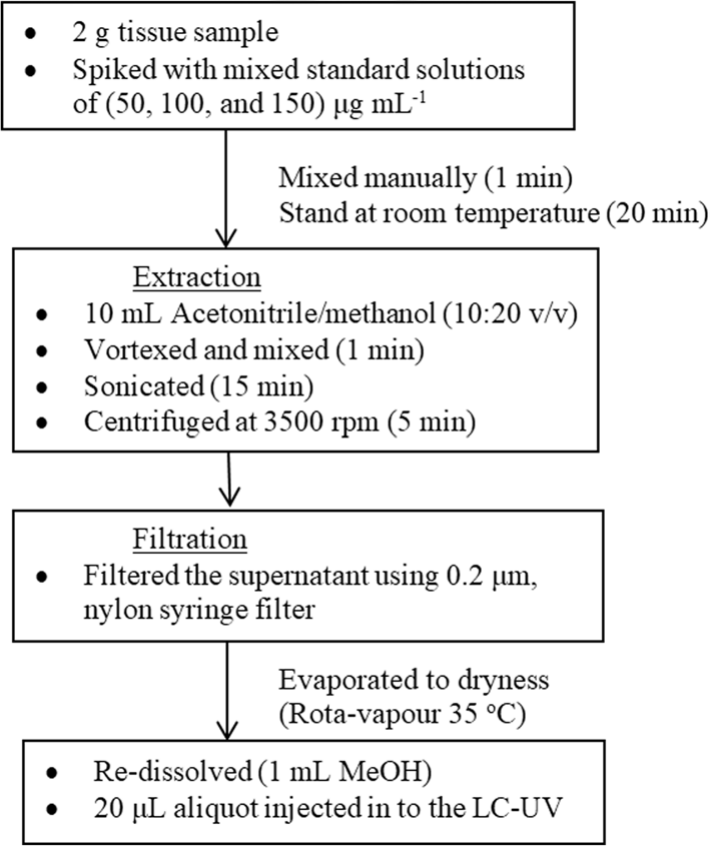
Flow chart of sample extraction for the determination of antibiotic residues in chicken tissue

The selected veterinary antibiotics were extracted from the chicken tissue using the method reported by Lopes et al. [31] and Bousova et al. [32] with a slight modification (Fig. 1). Chicken tissue samples were homogenized and ground in a blender with dry ice to obtain uniformed sample and kept at −20 °C. Two grams tissue sample was placed in 50 mL poly-propylene centrifuge tube and spiked with mixed standard solutions. The spiked sample was homogenised by manual shaking for 1 min and left to stand at room temperature for 20 min to allow the equilibration of the antibiotics with the chicken matrix before their extraction. In order to precipitate the proteins and extraction of analytes, 10 mL extraction solvent, acetonitrile/methanol (10:20 ratio v/v) were added to the mixture, homogenized via vortexing at 1000 rpm for 1 min and sonicated for 15 min. Then, the mixtures were centrifuged for 5 min at 3500 rpm, the top clear supernatant was transferred using syringe filter (0.2 μm, nylon) into 1 mL flask (Pyrex standard taper stopper), and evaporated to dryness using rotary evaporator at 35 °C. The obtained dried or concentrated residues were re-dissolved with 1 mL MeOH and 20 μL aliquot was injected in to the LC-UV system for analysis without filtration. All the analyses were carried out in triplicate.

Matrix-match (negative concentration) solutions were prepared from drug-free samples that have the same biological matrices as the real chicken tissue samples. Matrix-match solutions were used as the blank matrix for the method validation to validate the specificity of the method and to ensure or control that no carryover or matrix effects were present. The sample preparation procedures for negative samples were according to the method mentioned above without antibiotic standards.

### Parameter optimization

For the simultaneous detection method development, different factors that are affecting the separation processes were optimized. The parameters optimized include: pH, flow rate, organic mobile phases (types of solvent, concentration and composition), and the absorption wavelengths. The mixed standard solution was scanned in the wavelength region of 200–400 nm for proper separation. The effect of mobile phase on flow rate was also investigated using a different flow rate of 0.5, 1 and 1.2 mL min^−1^. The chromatographic parameters were evaluated by taking both the resolution and symmetry of the peaks into account.

## Results and discussion

### Selection of the type and volume of mobile phase

The chicken tissue was chosen for the method optimization and validation procedures because it is the most frequently consumed tissue with the highest content of proteins in the world [33]. Hence selection of appropriate mobile phase is an important step to get good separation. The mobile phase solvent type and composition was selected according to the physicochemical properties of antibiotic drugs (such as polarity, solubility, pK_a_ and miscibility with the aqueous phase) [29, 30]. The pK_a_ value is one of the main properties of an electrolyte that determines its chemical behaviour in solutions. Since most of the drugs are either weak acids or weak bases, they exist in both ionized and non-ionized forms depending on the pH of the solutions. Antibiotics are easily decomposed under strongly acidic or basic conditions by hydrolysis. Therefore, a suitable elution solvent should be carefully selected in order to achieve the highest recovery of the antibiotics contained in the tissue samples [34]. Therefore, preliminary experiments were performed using a hypersil BDS-C18 column (3 µm, 100 mm × 4 mm) for the selection of the type and volume of mobile phase.

Based on the reported literature, antibiotics have usually been separated on a reverse-phase column using acidic or basic mobile phases [35, 36]. The initial composition of the mobile phase was set at water-MeOH (75:25, v/v) to promote the retention of the most polar analytes. Then, due to the large number of analytes and their different affinities for the column, several elution programs and different ratios of water/acetonitrile with formic acid (0.1%) as eluent were tested for mobile phase efficiency of the target analytes under this study. The chromatograms obtained did not satisfactorily resolve all the signals. Resolution of the compounds was clearly affected by the acidity of the mobile phase. To prevent this, formic acid was replaced with phosphate buffer, and pH was adjusted to 8. A series of experiments were performed under the same experimental conditions and different types of phosphate buffers (i.e., sodium and potassium phosphate, citrate–phosphate buffer or McIlvaine buffer) in a different concentration and composition with methanol and acetonitrile. By taking consideration of both the resolution and symmetry of the peak into account, Na_2_HPO_4_ at the concentration of 0.05 M yielded the best resolution. Therefore, potassium phosphate and citrate–phosphate were ruled out and di-sodium phosphate was selected for further experiments.

To evaluate the effect of the volume and composition of organic solvents, a ratio of 30:30, 20:30, 15:25, and 10:20 v/v acetonitrile and methanol were studied. The ratios of acetonitrile to methanol volume at 10:20 in combination with 0.05 M Na_2_HPO_4_ give good resolution between analytes. Therefore, mobile phase comprised three solvent composition in a combination of 0.05 M Na_2_HPO_4_, acetonitrile and methanol (70:10:20) were found optimum for this experiment.

### Wavelength selection

**Fig. 2:**
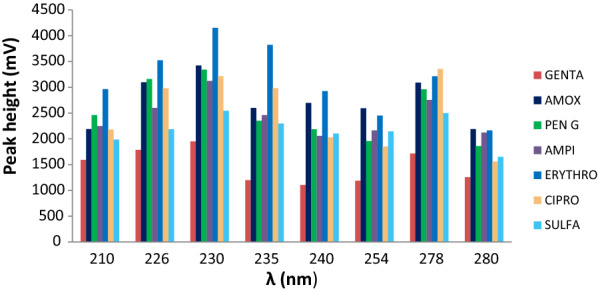
Detector response of seven selected antibiotic drugs analysed using eight different UV wavelengths (210, 226, 230, 235, 240, 254, 278 and 280) nm to enable selection of the optimal wavelength based on the optimized parameters, mobile phase: 0.05 Na_2_HPO_4_:ACN:MeOH (70:10:20), pH 8, temperature: 40 °C and flow rate: 1 mL min^−1^

The absorbance of a compound depends on the type of solvent, concentration and molar absorptivity (Beer’s law). The individual absorbance maxima for the targeted antibiotics ranged from 210 to 290 nm based on the literature review [37–40] but a fixed wavelength was used to monitor the mixed multi-component eluate. In mobile phase composition of 0.05 M Na_2_HPO_4_, acetonitrile and methanol (70:10:20), 20 μL mixed standards were injected at several UV wavelengths: 210, 226, 230, 235, 240, 254, 278 and 280 nm. From this investigation, the result showed that 230 nm yielded the largest overall relative peak height and peak area for all the analytes compared to those obtained at other wavelengths as can be seen in Fig. 2. The resulted maximum absorbance was different for each analyte. For example, three compounds (amoxicillin, ampicillin and penicillin G) give maximum UV absorption at 210 and 230 nm. The other four (erythromycin, gentamicin, sulfamethoxazole and ciprofloxacin) showed a good absorbance at 210, 226, 235 and 278 nm, respectively. However, Fig. 2 shows that erythromycin, gentamicin, ciprofloxacin, and sulfamethoxazole also have a maximum UV absorption at 230 nm comparatively with 210, 226, 235 and 278 nm which have maximum absorbance for each analytes. This is because the absorbance of a compound depends on the type of solvent, concentration, molar absorptivity and the effect of the other compounds present in the mixture. Therefore, 230 nm was selected as an optimum wavelength at which all the seven compounds showed good absorbance.

### Optimization of pH

**Fig. 3:**
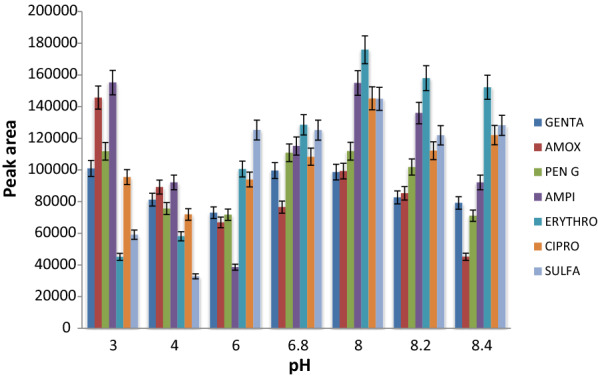
The effect of pH of the mobile phase to peak areas on the determination of analytes based on the optimized parameters, mobile phase: 0.05 Na_2_HPO_4_:ACN:MeOH (70:10:20), temperature: 40 °C, wavelength: 230 nm, and flow rate: 1 mL min^−1^

In the present work, studies involve only one variant at a time by keeping others as constant. The pH value is important as it affects the ionization status as well as the solubility of the analytes [41]. For efficient extraction of ionisable and relatively polar compounds, pH of the sample solution plays a key role. The pH of buffer or the mobile phase should be lower than two pH units below its pK_a_ value to obtain the target analytes in their unionized forms so that they have a higher tendency to partition into the organic phase [42]. In this study, the effect of pH was investigated by varying the pH from 3 to 8.4 using orthophsphoric acid and sodium hydroxide solution. The highest peak areas of the target antibiotics were obtained at pH 8 followed by a steady state in the range of (8–8.2) as can be seen in Fig. 3. Therefore, a mobile phase solution of pH 8 was chosen as the optimum extraction condition.

### Flow rate optimization

**Fig. 4:**
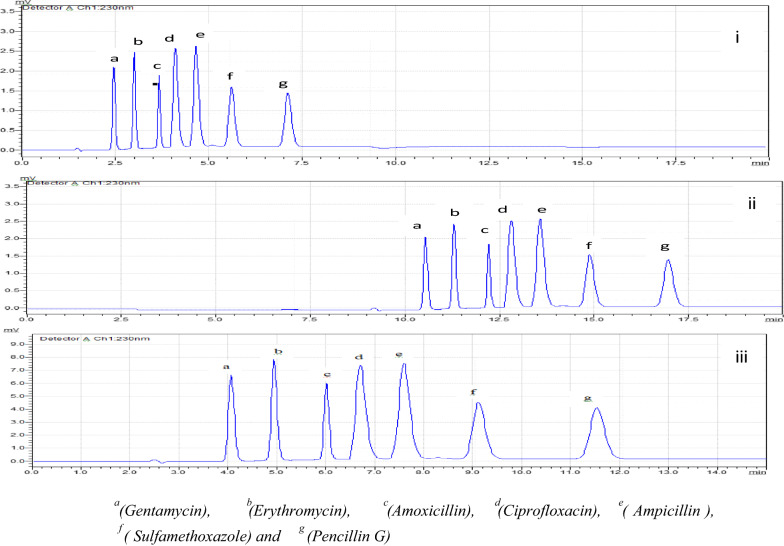
Chromatograms obtained using different mobile phase flow rates **i** 1.2 mL min^−1^, **ii** 0.5 mL min^−1^ and **iii** 1.0 mL min^−1^. The mixed standard chromatogram results found based on the optimized parameters, mobile phase: 0.05 Na_2_HPO_4_:ACN:MeOH (70:10:20), pH 8, temperature: 40 °C, wavelength: 230 nm, and flow rate: 1 mL min^−1^

The mobile phase speed for mixed standards were checked out at different flow rates (0.5–1.5) mL min^−1^ and the result obtained using 1 mL min^−1^ gave better resolution than others. This may be due to the interaction of the compound with the stationary and the mobile phase; a faster mobile phase flow limits the interaction of analyte with the stationary phase. It was observed that the increase in flow rate (1.2 mL min^−1^) decreases the retention time of all analyte compounds; and it adversely affected the resolution of some compounds. While decreasing the flow rate (0.5 mL min^−1^) increases the retention times and total run time, it caused and leads to broadening of the peaks and yielded poorly resolved peaks, in addition it is time consuming to get all peaks to appear, 17 min and more time is needed and more mobile phase amount consumed due to long time run. The optimum flow rate providing maximum sensitivity and the best analyte separation was 1.0 mL min^−1^ as shown in Fig. 4. Based on the above optimized conditions, the selected parameters that are suitable for the present work on method development were: mobile phase: 0.05 Na_2_HPO_4_: ACN:MeOH (70:10:20) (v/v/v), temperature: 40 °C, wavelength: 230 nm, and flow rate: 1 mL min^−1^.

### Column temperature

**Fig. 5:**
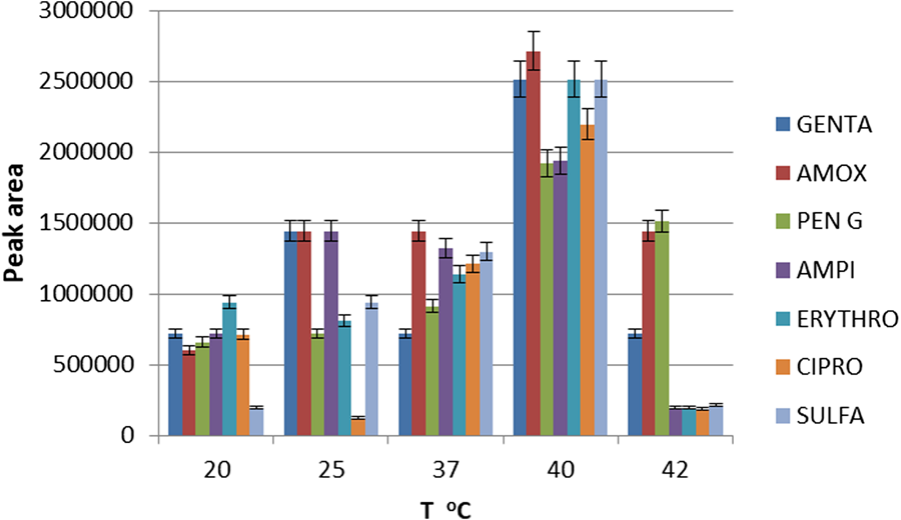
Detector response for column temperature variation, by keeping other variables constant and optimized, mobile phase: 0.05 Na_2_HPO_4_:ACN:MeOH (70:10:20), pH 8, wavelength: 230 nm, and flow rate: 1 mL min^−1^

The column temperature is an important parameter as it affects the stability status of antibiotics. Each antibiotic has different stability behavior at different temperatures, most antibiotic molecules decomposed or degraded with increases in temperature [43, 44]. The influences of the column temperature were investigated, by varying the temperature from 25 to 42 °C using LC solution software which controls the column temperature. At ambient temperature (25 °C), the responses were low for the selected components, as shown in Fig. 5, the column temperature at 40 °C was found to be the optimal setting, yielding the highest resolution, the greatest number of separated peaks and the strongest analyte response in combination with the above optimized mobile phase composition.

It should be noted that temperature can affect the separation of components. Many times this causes all of the analytes to come out sooner from the column, causing a reduction in the retention time [45, 46]. Changes in resolution are due to changes in peak separation and/or peak width. Decreasing column temperatures usually increase peak separation but often with a corresponding increase in peak width. If the increase in peak separation is greater than the increase in peak width, improved peak resolution occurs. Therefore, a proper column temperature control is essential for separations with marginal resolution of the critical peak pair. In most applications, antibiotic residue separation was done at about 40 °C to manage this significant effects from back pressure and temperature.

### Injection volume

In order to evaluate the maximum injection volume, some experiments were carried out by increasing the injection volume to 10, 20 and 50 μL. Injection volume 10 µL give small peak height. For larger injection volumes (50 μL), the more polar compounds did not show linearity of response with concentration, possibly due to the overload of the column. For example, gentamicin and erythromycin, which are more polar than the other compounds, did not show linearity response for larger injection volume 50 µL. Injection volume 20 μL was the optimum for this experiment and it is the maximum tolerable volume for the LC-UV system.

### Validation of the developed method

The developed method for the determination of selected antibiotics was validated according to the rules of the Commission Decision 2002/657/EC [28] that establish the validation guidelines and general and numeric criteria for evaluation of fitness of a method for residue analysis. The parameters were evaluated for, linearity, sensitivity, precision, accuracy, specificity, robustness, system suitability, CCα (decision limit) and CC*β* (detection capability) using both blank and spiked tissue samples at various concentrations.

### Standard calibration plots

**Fig. 6:**
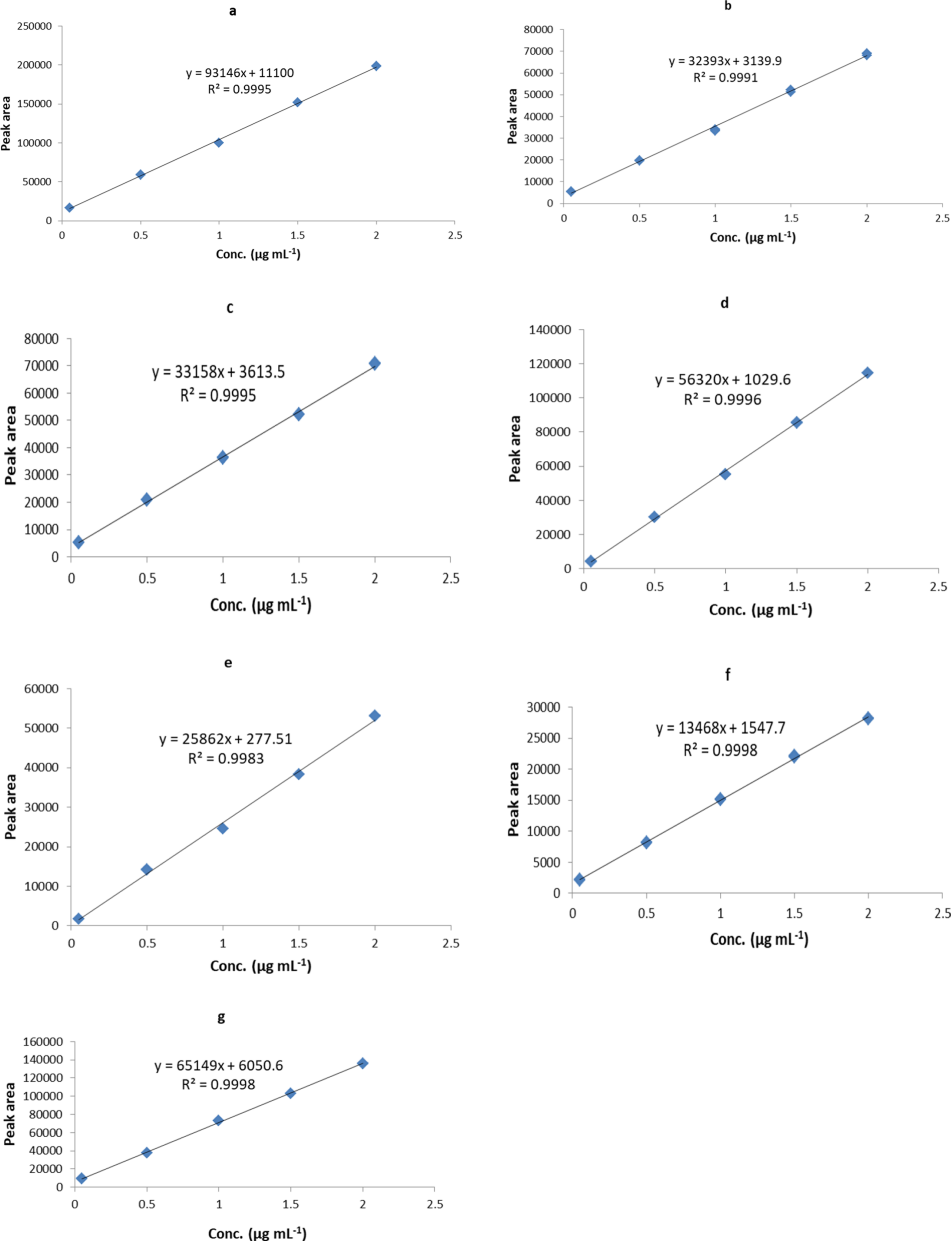
Calibration plots of the selected seven antibiotics with regression equation and correlation coefficient for each of the seven selected antibiotics based on the optimized parameters; mobile phase: 0.05 Na_2_HPO_4_:ACN:MeOH (70:10:20), pH:8, temperature: 40 °C, wavelength: 230 nm, flow rate: 1 mL min^−1^; **a** Gentamycin, **b** Erythromycin, **c** Amoxicillin, **d** Ciprofloxacin, **e** Ampicillin, **f** Sulfamethoxazole, **g** Penicillin G; The significance of independent factors was determined using Fisher’s statistical test for analysis of the variance (ANOVA) model

The calibration plots define the relationship between the detector response and the concentration of analyte in the sample matrix. For multiple analytes, a sample calibration plot was generated for each analyte [47]. The calibration plots were constructed by plotting the response ratio (ratio between peak area of antibiotic standards used on the x-axis and peak area of found concentration or response on the y-axis) in (μg mL^-1^). The calibration plot indicated a linear relationship between response ratio and antibiotic standard concentration with an acceptable correlation coefficient and regression parameters as summarized in Fig. 6. The method linearity was investigated in the concentration range of (0.05–150) μg mL^-1^. The linearity was studied for all the test antibiotics under optimised conditions and extended up to 300 μg mL^-1^ for erythromycin.

### Sensitivity

Calibration plots for each antibiotic with the respective correlation coefficient were calculated by least squares linear regression analysis of the peak area ratio of each analytes. The calculations for the limits of detection (LOD) were based on the standard deviation of the response and slope (S), of the calibration curve of antibiotic compounds y-intercepts of using the equation LOD = 3.3 × σ/S. Limits of quantitation (LOQ) were calculated by the equation LOQ = 10 × σ/S (Guidance for Industry Q2(R1), ICH, 2005) [48], where σ is the standard deviation of the response and S the slope of the calibration curve. The results are reported in Table 2 which shows that the LODs ranged from 0.098 to 0.255 μg mL^−1^ and the LOQs from 0.297 to 0.574 μg mL^−1^.

**Table 2:** Precision and sensitivity data of the seven examined antibiotics

ID	Antibiotics	LOD	LOQ	%Recovery	Precision in % RSD	
					Repeatability (% RSD, n = 3)	Reproducibility (% RSD, n = 3)
a	Gentamycin	0.146	0.442	99.1	1.1	1.1
b	Erythromycin	0.098	0.297	107	1.2	1.1
c	Amoxicillin	0.137	0.416	101	2.4	4.0
d	Ciprofloxacin	0.126	0.380	103	3.5	2.3
e	Ampicillin	0.255	0.774	104	4.7	4.2
f	Sulfamethoxazole	0.105	0.319	98.1	2.1	1.5
g	Pencillin G	0.189	0.574	98.6	4.2	4.6

### Selectivity

**Fig. 7:**
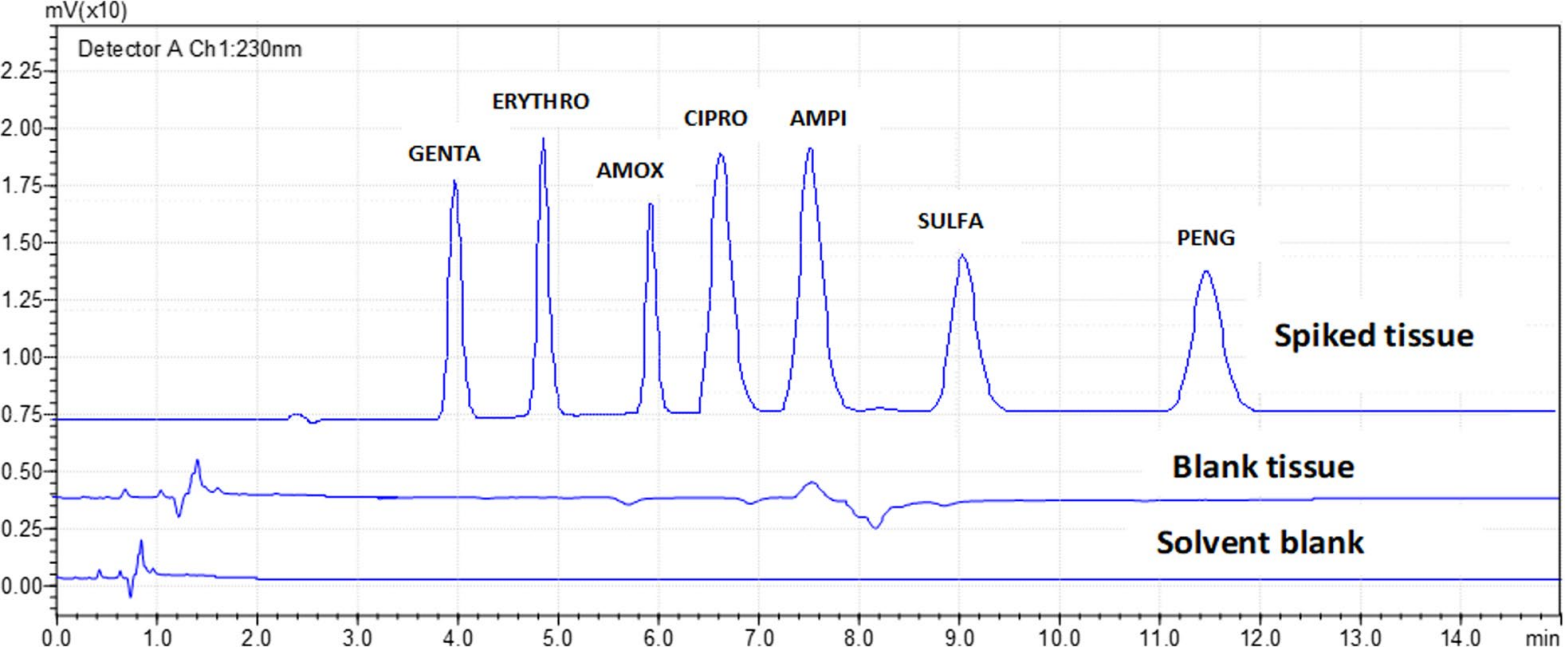
Overlay chromatograms of blank and spiked tissue samples based on the optimized parameters, mobile phase: 0.05 Na_2_HPO_4_:ACN:MeOH (70:10:20), pH 8, temperature: 40 °C, wavelength: 230 nm and flow rate: 1 mL min^−1^

The selectivity of the procedure in terms of the absence of interference compounds was checked by analysing drug-free and spiked samples of chicken tissue. The analysis was performed with optimized method and all the samples were checked for any interference at the retention times of the examined antibiotics at 230 nm. No endogenous compounds were found to interfere with examined antibiotics as shown in Fig. 7, the typical chromatograms of blank and spiked tissue samples have a good response and resolution for the targeted components of the selected seven antibiotics.

### Specificity

Specificity is the ability of the analytical method to distinguish between the analyte(s) and the other components in the sample matrix [49]. In order to investigate the specificity of the method for the interference components at the working wavelength, blank and matrix-match (drug-free) chicken tissue samples were scanned from 200 to 800 nm, the chromatograms are shown in Fig. 7. There was no interference peak observed in blanks and matrix-match tissue samples at the working wavelength of 230 nm. Therefore, the method presented in this study is specific for determination of the seven antibiotic compounds. Furthermore, non-interfering peaks appeared in the chromatogram of the spiked antibiotics retention times, the purities of the investigated peaks were all confirmed to be specific for the selected antibiotics on the optimized conditions.

### Precision

The precision of the method was evaluated in terms of repeatability (intra-day precision) and intermediate or reproducibility (inter-day precision). Repeatability was evaluated according to the matrix-matched approach by analyzing spiked drug-free chicken tissue samples and injected in triplicate on the same day, under the optimum conditions at three concentrations of 0.5, 1, and 1.5 times the permitted limit according to the European Decision (European Commission Decision 2002/657/EC) [28]. Ampicillin, amoxicillin, and pencillin G, have the MRL amount of 50 μg kg^−1^ and the spiked amount was (25, 50, 75) μg kg^−1^; ciprofloxacin, sulfamethoxazole, and gentamycin has MRL of 100 μg kg^−1^ and the spiked amount was (50, 100, 150) μg kg^−1^; erythromycin has MRL of 200 μg kg^−1^ and the spiked amount was (100, 200, 300) μg kg^−1^. The maximum residue limit (MRL) of antibiotics in the food of animal origin was found from commission regulation (EU) [17, 50].

Intermediate precision was evaluated using a similar procedure, but the samples were analysed on six consecutive days and in all cases by triplicate analysis. The measured peak areas were used to calculate the percent relative standard deviations (% RSDs) (Table 3). The result obtained for the precision study were regarded as acceptable for analysis, due to the small % RSDs that ranged from 0.6 to 7.6%, which were lower than the stipulated values of 15% [51].

**Table 3:** Intra-day precision, inter-day precision and recovery studies of the developed method for the determination of antibiotics in chicken tissue

Analyte	Spiked amount μg kg^−1^	Repeatability^a^ (n = 3 determinations)			Reproducibility^b^ (n = 3 determinations)		
		Measured ± SD (μg kg^−1^)	% RSD	Recovery (%)	Measured ± SD (μg kg^−1^)	% RSD	Recovery (%)
GENTA	50	48.0 ± 0.7	3.0	94.4	50.5 ± 2.5	2.1	101
	100	99.5 ± 2.1	1.1	99.0	99.8 ± 1.4	1.1	99.8
	150	149 ± 1.1	1.3	99.2	147 ± 1.2	1.1	99.0
AMOX	25	24.3 ± 0.9	3.7	97.2	24.8 ± 1.3	5.3	99.4
	50	50.5 ± 1.2	2.4	101	49.5 ± 2.0	4	99.0
	75	75.8 ± 1.1	1.4	101	75.7 ± 1.8	2.3	101
PEN G	25	24.9 ± 1.6	6.3	99.6	23.9 ± 1.8	7.5	95.6
	50	49.7 ± 2.1	4.2	99.4	50.3 ± 3.8	7.6	101
	75	75.3 ± 2.5	3.3	100	76.3 ± 3.0	3.9	102
AMPI	25	23.9 ± 2.0	3.3	96.0	23.5 ± 0.3	3.7	95.5
	50	50.9 ± 2.4	4.7	102	51.1 ± 2.1	4.2	102
	75	74.7 ± 2.8	1.3	101	74.9 ± 1.1	2.4	100
ERYTHRO	100	99.4 ± 1.7	1.1	99.4	98.9 ± 2.3	1.5	98.9
	200	200 ± 3.5	1.2	99.8	199 ± 3.4	1.1	99.3
	300	299 ± 3.9	0.9	99.8	299 ± 3.2	0.7	99.0
SULFA	50	47.0 ± 0.8	3.1	95.6	50.2 ± 3.5	2.4	101
	100	99.9 ± 2.3	2.1	98.9	99.4 ± 2.4	1.5	99.3
	150	149 ± 1.7	1.2	99.0	146 ± 1.6	1.3	97.0
CIPRO	50	50.7 ± 0.7	1.4	101	49.8 ± 1.0	2.1	99.6
	100	98.1 ± 3.2	3.5	98.7	101 ± 1.1	2.3	101
	150	144 ± 1.3	1.1	94.0	142 ± 1.3	1.4	95.0

^a^Studied by spiking the tissue samples on the same day, under the same experimental conditions

^b^Evaluated by spiking the indicated concentration levels in triplicate for six consecutive days

### Accuracy

To study the accuracy of the proposed method, recovery studies were carried out by applying standard addition at different levels in μg kg^−1^ to tissue sample. As reported in Table 3, the recoveries of the target compounds ranged from 94 to 102% for spiked tissue samples, which are within the acceptable range [51]. The result shows the optimized method was adequate for the simultaneous analysis of these antibiotics in practical chicken tissue samples.

### Robustness

Robustness is typically assessed by the effect of small deliberate changes to chromatographic methods on system suitability parameters such as peak retention, resolution, and efficiency [52] and provides an indication of its reliability during application. Robustness of the developed method was investigated after minor modifications of conditions including changes to the flow rate of the mobile phase 0.8 and 1.2, variations to pH of mobile phase between 7.9 and 8.1, and analysis temperature between 38 and 42 °C. The results in Table 4 revealed that the developed method is robust, and the peaks are well separated and elute with acceptable symmetry and resolution.

**Table 4:** Effects of the analytical parameters change performed for robustness evaluation in flow rate, pH of mobile phase and temperature

Compounds	Parameters changed for robustness study									
	Flow rate (mL min^−1^)				pH of mobile phase			Analysis temperature °C		
		0.8	1.0	1.2	7.8	8.0	8.2	38	40	42
GENTA	Peak symmetry	0.96	1.00	0.99	0.59	0.47	0.22	0.22	0.90	0.84
	% RSD	1.14	3.33	2.15	1.96	1.96	3.02	2.43	3.10	3.10
	Rt (min)	3.78	3.85	3.65	3.80	3.86	3.88	3.56	3.83	3.79
AMOX	Peak symmetry	0.96	0.94	0.79	0.97	0.78	1.00	0.94	0.39	0.68
	% RSD	3.27	2.54	3.70	0.22	1.24	4.32	2.37	2.88	1.25
	Rt (min)	5.89	5.82	5.90	5.69	5.81	5.76	5.70	5.89	5.79
PEN G	Peak symmetry	0.74	0.25	0.86	0.85	0.64	0.94	0.95	1.59	0.65
	% RSD	4.57	3.26	2.37	2.15	1.28	0.26	1.24	1.24	0.27
	Rt (min)	11.65	11.25	11.28	11.98	11.36	11.98	11.22	11.21	11.14
AMPI	Peak symmetry	0.52	1.00	0.50	1.00	1.26	0.78	1.46	0.68	0.63
	% RSD	2.43	3.27	1.25	5.32	4.24	6.32	1.24	4.24	1.98
	Rt (min)	7.37	7.38	7.46	7.21	7.31	7.21	7.97	7.33	7.30
ERYTHRO	Peak symmetry	1.25	0.95	0.95	1.15	1.09	1.74	0.22	0.84	0.68
	% RSD	1.24	1.42	1.98	2.37	4.37	4.98	2.40	1.87	1.79
	Rt (min)	4.75	4.75	4.80	4.72	4.71	4.75	4.69	4.76	4.60
SULFA	Peak symmetry	0.99	0.99	1.00	1.00	0.61	0.67	0.42	0.90	0.47
	% RSD	3.02	2.67	4.14	2.43	3.10	3.10	3.27	1.24	0.24
	Rt (min)	8.80	8.82	8.86	8.81	8.75	8.12	8.54	8.85	8.13
CIPRO	Peak symmetry	0.91	0.59	0.22	1.04	1.00	0.91	0.91	0.59	0.22
	% RSD	3.04	1.32	3.26	2.65	3.12	2.37	1.24	3.26	3.01
	Rt (min)	6.50	6.55	6.24	6.56	6.70	6.28	6.45	6.12	6.25

### System suitability

The system suitability test of a chromatographic method is used to ensure the chromatographic system is adequate for application to samples. The parameters considered for this test includes retention time, resolution (to the adjacent peak), peak symmetry and number of theoretical plates [53]. These parameters were investigated using the optimized chromatographic conditions. The results met the acceptance criterion as listed in Table 5 and reflect good performance for all the selected analytes.

**Table 5:** System suitability results determined for the developed chromatographic method

Compound	Retention time (min)	Resolution	Peak symmetry	Theoretical plates (N)
Gentamycin	3.851	2.39	0.986	112,263
Erythromycin	4.752	8.52	0.875	214,489
Amoxicillin	5.822	3.69	0.884	152,353
Ciprofloxacin	6.554	3.25	0.657	321,443
Ampicillin	7.382	2.98	0.793	432,008
Sulfamethoxazole	8.824	15.23	0.642	122,443
Pencillin G	11.252	14.36	0.527	413,963
Reference values [53]		> 1.5	> 0.50	> 13,333 (2000/column)

### Decision limit (CCα) and detection capability (CCβ)

The decision limit and detection capability correspond to the regulation of the European Commission 2002/657/EG [28]. CCα (decision limit) is a non-conformity of the samples concluded, with an error probability *α* of 5%, while CCβ (detectability) is defined as the smallest detectable content of the substance and/or quantified in a sample with an error probability β of 5% (1% for prohibited substances). CCα is calculated by analyzing blanks spiked with the analyte at the MRL or by using the calibration curve procedure in accordance with ISO 11843 from the data obtained during the validation of the method. CCβ is calculated from the CCα value and the standard deviation at that concentration. For substances with MRLs, the decision limit and detection capability must be greater than the MRL, the values of α and β errors must be less than or equal to 5%.

The determination of these parameters was obtained by the analysis and extraction of five blank samples spiked at levels of concentration at their MRL level. In order to complete the validation procedure for tissue samples, the decision limit CCα (α = 5%) were calculated as the mean values of the found concentrations at the permitted limit plus 1.64 times the corresponding standard deviations. The detection capability (CCβ) (β = 5%) was obtained by adding CCα values to 1.64 times the corresponding standard deviation of spiked tissue samples. Table 6 summarizes the obtained CCα and CC*β* value for chicken tissue at their MRL level.

**Table 6:** The values of (decision limit) CCα and (detection capability) CCβ for chicken tissue at the MRL enacted by the EU

Analyte	Added (μg kg^−1^)	Found ± SD (μg kg^−1^)	Error α (1.64 × SD)	CCα (μg kg^−1^)	Found ± SD (μg kg^−1^)	Error β. (1.64 × SD)	CCβ (μg kg^−1^)
GENTA	100	99.5 ± 2.1	3.44	103	99.8 ± 1.4	2.30	106
AMOX	50	50.5 ± 1.2	1.97	52.0	49.5 ± 2.0	3.28	55.3
PEN G	50	49.7 ± 2.1	3.44	53.4	50.2 ± 2.1	3.44	56.9
AMPI	50	50.9 ± 2.4	3.94	53.9	51.1 ± 2.1	3.44	57.4
ERYTHRO	200	200 ± 3.0	4.92	205	199 ± 2.4	3.94	209
SULFA	100	99.9 ± 2.3	3.77	104	99.4 ± 2.4	3.94	108
CIPRO	100	98.1 ± 2.2	3.61	104	101 ± 1.1	1.80	105

SD = standard deviation

### Application of the method to real samples

The method was developed and optimized using chicken tissue matrix-match samples which have the same biological matrix but without analyte. However in order to prove its applicability and to make the method suitable for performing routine analyses, it was further applied in real tissues and the organ meat of chickens (kidney and liver). Chicken samples purchased from five local supermarkets were analyzed for their antibiotic residue using the developed and validated method. Most of the samples analyzed were free from the target analytes, except for chicken samples from one supplier. In two chicken samples purchased from one supermarket, amoxicillin, ampicillin, penicillin G and sulfamethoxazole were detected in muscle tissue and organ meats at levels below established MRLs. Although the amounts detected were below limits of quantification, chicken organ meats (kidney and liver) observed to present consistently higher values of the detected analytes in comparison to the other tested muscle tissues. Ciprofloxacin, gentamycin and erythromycin were not detected in all the chicken samples.

## Conclusion

A simple, accurate, precise and robust liquid chromatography with UV detection (LC-UV) method has been developed for the simultaneous determination of seven selected multi-residue, multiclass drugs in the chicken tissue using a single optimized condition. Chicken tissue was analyzed for the residues of seven antibiotic residues including gentamicin amoxicillin, ampicillin, ciprofloxacin, erythromycin, penicillin G and sulphamethoxazole. The developed method was validated using European Commission Decision 2002/657/EC guidelines, which proves the reliability of the proposed method. The accuracy of the method was validated by percentage recovery and found to be in the acceptable range. Analytical method development and validation are continuous and interconnected activities. The developed analytical method has many advantages; it has simple sample preparation procedure based on acetonitrile extraction of antibiotics in the food of animal origin, cost effective with less time separation, i.e., 14 min chromatographic run which allowed seven multiclass antibiotic residues analyses to be performed within one injection volume. The method validation parameters demonstrate its reliability, satisfactory recovery, precision, and good specificity provided good performance that was easily applied to the analysis of multiclass multi-residue analysis in chicken samples at µg mL^−1^ levels. Furthermore, to the best of our knowledge, this is the first instance in which an analytical procedure for the simultaneous determination of these selected seven multiclass, multi-residue analysis using LC-UV from chicken tissue samples. This is a cost-effective and alternative with simple instrumentation approach for laboratories that lack highly specialized state-of-the-art instrumentation.

## Data Availability

The data sets used and analyzed during the study are available to readers as in the manuscript. There are no additional data with the authors. All the data are included in the manuscript.

## Abbreviations

AA: Addis Ababa
ADI: Acceptable daily intake
AMOX: Amoxicillin
AMPI: Ampicillin
ANOVA: Analysis of variance
CAC: Codex Alimentarius Commission
ACN: Acetonitrile
CCα: Decision limit
CC*β*: Detection capability
CIP: Ciprofloxacin
EFDA: Ethiopian Food and Drug Administration
EPHI: Ethiopian Public Health Institute
ERYTHRO: Erthyromycin
FAO: Food and Agriculture Organization
GC: Gas chromatography
GENTA: Gentamicin
HPLC: High performance liquid chromatography
HR-MS: High resolution-mass spectrometry
ICH Q2(R1): International Conference on Harmonisation for Registration of Pharmaceuticals for Human Use
LC–MS/MS: Liquid chromatography–tandem mass spectrometry
LC-QToFMS: Liquid chromatography-quadrupole time-of-flight mass spectrometry
(LC-QqQ-MS): Liquid chromatography-triple quadrupole-mass spectrometry
LC-UV: Liquid chromatography with ultraviolet detection
LOD: Limits of detection
LOQ: Limits of quantification
MRLs: Maximum residue limits
PEN G: Penicillin G
RSD: Relative standard deviation
SULFA: Sulfamethoxazole
WHO: World Health Organization
